# Propensity matched analysis of DPA or DPL used within the first hour for severely hypotensive blunt trauma patients

**DOI:** 10.1016/j.sopen.2025.01.005

**Published:** 2025-01-25

**Authors:** Mallory Jebbia, Jeffry Nahmias, Sebastian Schubl, Matthew Dolich, Michael Lekawa, Allen Kong, Areg Grigorian

**Affiliations:** University of California, Irvine, Department of Surgery, Division of Trauma, Burns and Surgical Critical Care, Orange, California, USA

**Keywords:** Diagnostic peritoneal lavage, Diagnostic peritoneal aspiration, Blunt trauma, Severe hypotension, Hemorrhagic shock

## Abstract

**Background:**

Prior single-center reports advocate for use of diagnostic peritoneal aspiration or lavage (DPA/DPL) to identify blunt trauma patients (BTPs) with intra-abdominal hemorrhage who require emergent surgery. Despite this, concerns exist over the potential for DPA/DPL to delay transfer to the operating room (OR). We hypothesized that DPA/DPL application in severely hypotensive BTPs would lead to increased OR transfer time and in-hospital mortality.

**Methods:**

The 2017–2019 TQIP database was queried for adult BTPs presenting with severe hypotension (systolic blood pressure <70 mmHg) who underwent any operative intervention within two-hours. Using a 1:2 propensity-score model, patients who underwent DPA/DPL within one-hour of arrival were compared with those who did not, controlling for age, sex, comorbidities, ≥6 units of packed red cells within 4 h, and injury profile.

**Results:**

From 5514 patients, 62 (1.1 %) underwent DPA/DPL. We matched 52 DPA/DPL patients to 104 patients not undergoing DPA/DPL. There were no differences in the matched variables between cohorts (all p > 0.05). Compared to those not undergoing DPA/DPL, patients undergoing DPA/DPL had a higher rate/risk of in-hospital complications (59.6 % vs. 39.4 %, p = 0.02) (OR 2.27, CI 1.15–4.47, p = 0.02) but statistically similar rate/risk of death (65.4 % vs. 50.0 %, p = 0.07) (OR 1.89, CI 0.95–3.76, p = 0.07). Time to OR was similar between both groups (DPA/DPL 39 min vs. non-DPA/DPL 42 min, p = 0.87).

**Conclusion:**

DPA or DPL used within the first hour of arrival does not appear to delay time to OR and does not increase risk of death. This challenges concerns over potential DPA/DPL-associated delays and heightened mortality risks.

## Background

Diagnostic peritoneal lavage (DPL) was first described in 1965 as the primary method for detecting hemorrhage in the abdominal cavity after trauma [1]. The technique involves placement of a needle into the peritoneal cavity inferior to the umbilicus, advancement of a guidewire, placement of an 8F catheter over the wire, infusion of 1 l of saline, and subsequent aspiration of this fluid for analysis of blood or bile. This was often used in hypotensive blunt trauma patients in whom an abdominal exam was unreliable, due to altered mental status or spinal cord injury, when ultrasound was not available or equivocal [2]. Since it is typically done emergently in the trauma bay, the trauma surgeon is oftentimes performing this procedure.

The inception of computed tomography (CT) in 1971 and its subsequent technological advancement have significantly decreased the reliance on DPL [3]. Early comparative analyses in the 1980s favored DPL for its sensitivity and specificity in diagnosing intra-abdominal bleeding over CT [4,5]. However, as CT imaging quality enhanced during the 1990s, studies began to report a rise in non-therapeutic laparotomies following DPL compared to CT [6]. Concurrently, a paradigm shift occurred in the management of solid organ injuries, transitioning from operative to non-operative approaches for hemodynamically stable patients [7]. This shift was facilitated by CT's ability to offer more detailed anatomical information, influencing surgical decision-making beyond the mere presence of blood as indicated by DPL.

Despite technological advancements, there remains some advocacy for diagnostic peritoneal aspiration or lavage (DPA/DPL) in certain clinical settings, especially in the context of blunt trauma patients (BTPs) where rapid identification of intra-abdominal hemorrhage is crucial for determining the need for emergency surgery [[8], [9], [10], [11]]. However, the use of DPA/DPL in this context may potentially delay transfer to the operating room (OR). Thus, we hypothesized that the application of DPA/DPL in severely hypotensive BTPs may prolong OR transfer times and increase in-hospital mortality.

## Methods

The Trauma Quality Improvement Program (TQIP) database was utilized for this study. Given that it is a deidentified national database, the study was deemed exempt by our institutional review board. The 2017–2019 TQIP database was queried for adults (≥18 years) undergoing any emergent operation (within 2 h of arrival) after blunt trauma and who presented to the trauma bay with severe hypotension, defined as a systolic blood pressure (SBP) <70 mmHg. The primary outcome was utilization of DPA/DPL within 2 h.

Demographic data points that were collected included age, sex, and comorbidities including congestive heart failure (CHF), chronic obstructive pulmonary disease (COPD), myocardial infarction (MI), cerebrovascular accident (CVA), peripheral arterial disease (PAD), chronic kidney disease, smoking, and steroid use. The injury profile included the injury severity score (ISS), abbreviated injury scale (AIS) of body regions and specific injuries such as traumatic brain injury, thoracic injury, solid organ and hallow viscous injuries, as well as extremity and spine fractures. Other vitals on arrival including heart rate (HR), and respiratory rate (RR) were also recorded.

Due to the observed imbalance in the sample size between patients that underwent DPA/DPL within 2 h and those that did not, we performed a 1:2 propensity matched model. The propensity score used in our analysis was derived from a logistic regression model in which the dependent variable was the utilization of DPA/DPL. The variables we used in our model included age, sex, comorbidities, ≥6 units of packed red cells within 4 h, and injury profile. These factors were chosen as they have been shown to be significant predictors of mortality in blunt trauma patients [[12], [13], [14]]. Patients with similar propensity scores were matched in a 1:2 ratio to compare outcomes among those undergoing [(+)DPA/DPL] and those that did not [(−)DPA/DPL]. We included in our analysis only those cases that were within 0.001 of the estimated logit. This technique of defining the closeness of a matched case is termed caliper matching and is the validated method of emulating randomization in observational studies [12]. Once propensity scores were calculated for each case, one (+)DPA/DPL patient and two matched (−)DPA/DPL patients were removed from the sample. If a (+)DPA/DPL patient did not have a close match available, they were excluded from any further analysis.

The primary outcome measured was mortality. A secondary outcome was time to OR. Other measured outcomes included in-hospital complications such as unplanned intubation, unplanned return to the operating room, pneumonia, acute respiratory distress syndrome (ARDS), organ space surgical site infection (SSI), superficial incision SSI, deep SSI, catheter associated urinary tract infection (CAUTI), central line associated blood stream infection (CLABSI), osteomyelitis, sepsis, cardiac arrest, cerebrovascular accident (CVA), deep venous thrombosis, pulmonary embolism, myocardial infarction, extremity compartment syndrome, pressure ulcer and acute kidney injury. Other outcomes evaluated were intensive care unit (ICU) admission and ICU length of stay (LOS).

All analyses were performed with IBM SPSS Statistics for Windows (Version 29, IBM Corp., Armonk, NY). A Mann-Whitney-*U* test was used to compare continuous variables and a chi-square was used to compare categorical variables in the bivariate analysis. Categorical data was presented as percentages while continuous data was presented as a median with interquartile range. P-values were defined as statistically significant if <0.05.

## Results

Of 5514 blunt trauma patients undergoing an emergent operation and with severe hypotension on arrival, 62 (1.1 %) underwent DPA/DPL. We matched 52 (+)DPA/DPL patients to 104 (−)DPA/DPL patients. There were no differences in the matched variables between cohorts (all p > 0.05). The median age of patients in the (+)DPA/DPL group was 50, the most common comorbidities were hypertension and substance use disorder (each 13.5 %) and the most common injuries were to the spine (59.6 %) and ribs (53.8 %). (Table 1, Table 2).

**Table 1:** Demographics of operative patients undergoing DPA/DPL within 1 h vs. no DPA/DPL.

Characteristic	No DPA/DPL	DPA/DPL	p-Value
	(n = 104)	(n = 52)	
Age, year, median (IQR)	51 (30)	50 (31)	0.395
Male, n (%)	70 (67.3 %)	35 (67.3 %)	1.000
ISS 15, n (%)	96 (92.3 %)	48 (92.3 %)	1.000
Vitals on arrival, n (%)			
Hypotensive (SBP < 90)	54 (54.5 %)	31 (62.0 %)	0.385
Tachypneic (>22)	40 (42.1 %)	12 (24.5 %)	0.037
Tachycardic (>120)	25 (25.0 %)	19 (36.5 %)	0.137
GCS ≤8	55 (54.5 %)	34 (65.4 %)	0.194
Comorbidities, n (%)			
Hypertension	14 (13.5 %)	7 (13.5 %)	1.000
CHF	2 (1.9 %)	1 (1.9 %)	1.000
Smoking	13 (12.5 %)	5 (9.6 %)	0.595
COPD	2 (1.9 %)	1 (1.9 %)	1.000
Diabetes	10 (9.6 %)	4 (7.7 %)	0.692
CKD	2 (1.9 %)	0 (0 %)	0.314
Cirrhosis	2 (1.9 %)	1 (1.9 %)	1.000
Substance abuse	7 (6.7 %)	7 (13.5 %)	0.166
Psychiatric disorder	6 (5.8 %)	5 (9.6 %)	0.376

IQR = interquartile range, ISS = injury severity score, SBP = systolic blood pressure, GCS = Glasgow coma scale, OR = operating room, CHF = congestive heart failure, COPD = chronic obstructive pulmonary disease, CKD = chronic kidney disease.

**Table 2:** Injuries in operative patients undergoing DPA/DPL within 1 h vs. no DPA/DPL.

Injury n, (%)	No DPA/DPL	DPA/DPL	p-Value
	(n = 104)	(n = 52)	
Severe injury^a^, n (%)			
Head	32 (30.8 %)	16 (30.8 %)	1.000
Thorax	42 (40.4 %)	21 (40.4 %)	1.000
Abdomen	34 (32.7 %)	17 (32.7 %)	1.000
Traumatic brain injury	46 (44.2 %)	25 (48.1 %)	0.649
Skull fracture	44 (42.3 %)	20 (38.5 %)	0.645
Heart	12 (11.5 %)	3 (5.8 %)	0.249
Rib fracture	63 (60.6 %)	28 (53.8 %)	0.421
Lung	43 (41.3 %)	26 (50.0 %)	0.305
Diaphragm	6 (5.8 %)	7 (13.5 %)	0.101
Spleen	32 (30.8 %)	18 (34.6 %)	0.627
Pancreas	9 (8.7 %)	2 (3.8 %)	0.269
Stomach	1 (1.0 %)	3 (5.8 %)	0.073
Small intestine	11 (10.6 %)	7 (13.5 %)	0.595
Colon	12 (11.5 %)	9 (17.3 %)	0.320
Kidney	9 (8.7 %)	7 (13.5 %)	0.351
Pelvic fracture	37 (35.6 %)	17 (32.7 %)	0.721
Cervical fracture	16 (15.4 %)	13 (25.0 %)	0.146
Spine fracture	53 (51.0 %)	31 (59.6 %)	0.307
Cervical cord	4 (3.8 %)	1 (1.9 %)	0.520
Spinal cord	10 (9.6 %)	3 (5.8 %)	0.413
Upper extremity fracture	22 (21.2 %)	16 (30.8 %)	0.187
Lower extremity fracture	37 (35.6 %)	26 (50.0 %)	0.084

a Severe injury was defined by the abbreviated injury scale.

There were no differences in the types of operations performed between the two groups (p > 0.05). The most common organ system operated on was the gastrointestinal tract (74 cases total [47.4 %]). Compared to the (−)DPA/DPL group, patients in the (+)DPA/DPL group had a similar time to OR (39 min vs. non- 42 min, p = 0.87) but a higher rate of overall complications (59.6 % vs. 39.4 %, p = 0.017) and a statistically similar mortality rate (65.4 % vs. 50.0 %, p = 0.07). Stroke was seen in 3.8 % of (+)DPA/DPL patients while no strokes were seen in the (−)DPA/DPL group (p = 0.044) (Table 3). There was no statistically significant difference in LOS between the two groups ((+)DPA/DPL 2 days vs (−)DPA/DPL 6 days, p = 0.351). On univariable logistic regression, the risk of complications (OR 2.27, CI 1.15–4.47, p = 0.02) and death (OR 1.89, CI 0.95–3.76, p = 0.07) were similar (Table 4).

**Table 3:** Clinical outcomes in operative patients undergoing DPA/DPL within 1 h vs. no DPA/DPL.

Outcome	No DPA/DPL	DPA/DPL	p-Value
	(n = 104)	(n = 52)	
LOS, days, median (IQR)	6.0 (19)	2.0 (18)	0.351
Time to OR (minutes), median (IQR)	42.0 (37.2)	38.5 (38.4)	0.865
Type of operation, n (%)			
Gastrointestinal	48 (46.2 %)	26 (50.0 %)	0.650
Respiratory	30 (28.8 %)	18 (34.6 %)	0.462
Hepatopancreaticobiliary	14 (13.5 %)	13 (25.0 %)	0.073
Urinary	36 (34.6 %)	20 (38.5 %)	0.637
Cardiovascular	17 (16.3 %)	11 (21.2 %)	0.461
Complications, n (%)	41 (39.4 %)	31 (59.6 %)	0.017
Stroke/CVA	0 (0 %)	2 (3.8 %)	0.044
Cardiac arrest	23 (22.1 %)	22 (42.3 %)	0.009
Myocardial infarction	0 (0 %)	1 (1.9 %)	0.156
Pneumonia/VAP	2 (1.9 %)	4 (7.7 %)	0.077
ARDS	1 (1.0 %)	2 (3.8 %)	0.216
Unplanned intubation	5 (4.8 %)	1 (1.9 %)	0.377
Unplanned return to OR	5 (4.8 %)	2 (3.9 %)	0.803
Unplanned ICU admission	5 (4.8 %)	0 (0 %)	0.108
Deep SSI	2 (1.9 %)	3 (5.8 %)	0.199
Organ space SSI	0 (0 %)	1 (1.9 %)	0.156
CAUTI	2 (1.9 %)	0 (0 %)	0.314
CLABSI	2 (1.9 %)	1 (1.9 %)	1.000
Acute kidney injury	3 (2.9 %)	1 (1.9 %)	0.720
DVT	7 (6.7 %)	2 (3.8 %)	0.466
Mortality, n (%)	52 (50.0 %)	34 (65.4 %)	0.069

LOS = length of stay, IQR = interquartile range, OR = operating room, CVA = cerebrovascular accident, VAP = ventilator associated pneumonia, ARDS = acute respiratory distress syndrome, ICU = intensive care unit, SSI = surgical site infection, CAUTI = catheter associated urinary tract infection, CLABSI = central line associated blood stream infection, DVT = deep vein thrombosis.

**Table 4:** Multivariable^a^ logistic regression analyses for operative patients undergoing DPA/DPL within 1 h vs. no DPA/DPL.

Risk factor	OR	CI	p-Value
Risk of mortality:			
DPA/DPL vs no DPA/DPL	1.889	0.949–3.761	0.070
Risk of complications:			
DPA/DPL vs no DPA/DPL	2.268	1.150–4.474	0.018

a Controlled for age, injury severity score, vitals on arrival, severe abbreviated injury scale for the head/thorax/abdomen, and comorbidities (cirrhosis, chronic obstructive pulmonary disease, functional status, and chronic kidney disease).

## Discussion

Contrary to previous concerns, our findings indicate that DPL does not significantly delay OR transfer or increase mortality in hypotensive BTPs. While CT remains the standard for stable patients due to its detailed anatomical assessment capabilities, the potential value of DPA/DPL in unstable patients can be important in certain clinical scenarios, particularly multicavity hemorrhage. This challenges the prevailing perception that DPA/DPL is an antiquated procedure with no role in modern day trauma care.

The role of DPA/DPL in contemporary trauma care is not well-defined. While CT is the preferred method for stable patients because of its detailed anatomical assessment capabilities, DPA/DPL may still hold significant value for unstable patients in specific clinical situations. Its potential utility is most appreciated in blunt trauma patients with multiple possible sources of hemorrhage, where the trauma surgeon must decide which cavity to address first, or whether surgical intervention is necessary at all. Schellenberg et al. describe two subgroups of blunt trauma patients in whom DPA/DPL is particularly useful: the unstable blunt trauma patient with an equivocal or negative FAST exam, or the unstable blunt trauma patient with a known history of cirrhosis and a positive FAST exam. They go on to suggest that DPA/DPL in contemporary practice can be an adjunct to the FAST exam in unstable blunt trauma patients [13]. In fact, Cha et al. found that DPL had a higher sensitivity for abdominal injury in the hemodynamically unstable blunt trauma patient compared to FAST [14]. The similar times to OR between the two patient cohorts in our study suggest that DPA/DPL can be integrated into the acute management of severely hypotensive blunt trauma patients without detrimental time delays.

Similar to the argument that DPA/DPL can serve as an adjunct to the FAST exam, one could also argue for its utility alongside CT. Gonzalez et al. found that using DPL in conjunction with CT reduced the rate of non-therapeutic laparotomies after blunt trauma [15]. However, this relies on a patient being stable enough to get a CT scan, which is not always the case. While the exact role of DPA/DPL may have changed over the years with the advent of more advanced CT technology, it does not appear that there is no role for the procedure. Specifically, our study suggests DPA/DPL remains a valid option in unstable blunt trauma patients as it does not delay time to the OR or increase mortality.

With the evolution of CT scans, we have seen a decline in the use of DPA/DPL and a corresponding decrease in training the next generation of surgeons in these procedures. Rhodes et al. found that despite rising numbers of blunt trauma patients over the years, the use of DPA/DPL continues to decrease [16]. Similar studies have shown a decline in utilization of the technique, and some have gone as far as to suggest trauma guidelines be revised to stop teaching the use of DPA/DPL to students [17]. This sentiment seems to be echoed in surgical trainees in the UK as Bhan et al. found that 33 % of trainees found DPA/DPL to be obsolete. However nearly 40 % reported they had never even observed a DPA/DPL, yet they would consider using the technique if CT was unavailable [18]. With trainees reporting they have not seen or performed DPA/DPL in training, the next question one might ask is if DPA/DPL leads to more complications directly related to performing the procedure. Our study found no difference in surgical site or deep space infections, suggesting that DPA/DPL was done in a safe manner without cause of injury. This is mirrored in several studies showing DPA/DPL are safe procedures [19,20].

This study did find that in-hospital complication rates were overall higher in the DPA/DPL group. Specifically, there were higher rates of cerebrovascular accidents and cardiac arrests in the DPA/DPL group. Despite the increased cardiac arrest rate in the DPA/DPL group, there was no difference in mortality rate between the two groups. The increase in cardiac arrest rate in the DPA/DPL group could be related to an increased rate of blunt cardiac injury which is more difficult to diagnose. Additionally, the cardiac arrest could have occurred much after the index operation and may have been attributed to other concurrent complications.

This study has limitations inherent in a database study. Because it is retrospective it is subject to selection bias and missing or misclassified data. Additionally, as a nationwide study, it aggregates data from a variety of healthcare settings across the country, each with its own unique protocols and slight variations in trauma practice. Therefore, the trends observed may not reflect the experience at every individual institution. The use and results of a FAST exam are also not readily available in the TQIP dataset. Finally, the database lacks specific details such as who got a CT scan, or details about the patients' response to blood products which could further elucidate the patient's overall status and need for operative intervention.

## Conclusion

DPA or DPL used within the first hour of arrival does not appear to delay time to OR and does not increase risk of death. This challenges concerns over potential DPA/DPL-associated delays and heightened mortality risks.

## CRediT authorship contribution statement

**Mallory Jebbia:** Writing – review & editing, Writing – original draft. **Jeffry Nahmias:** Writing – review & editing, Conceptualization. **Sebastian Schubl:** Writing – review & editing. **Matthew Dolich:** Writing – review & editing. **Michael Lekawa:** Writing – review & editing. **Allen Kong:** Writing – review & editing. **Areg Grigorian:** Supervision, Formal analysis.

## Ethical approval statement

This study was conducted in accordance with the ethical standards of the institutional review board (IRB). The IRB deemed this project exempt from review given only de-identified data was utilized.

## Funding

There is no funding to report for this project.

## Declaration of competing interest

The authors report no conflicts of interest.
